# Comparative Pharmacokinetics of Meloxicam Between Healthy Post-partum vs. Mid-lactation Dairy Cattle

**DOI:** 10.3389/fvets.2020.00548

**Published:** 2020-09-08

**Authors:** Rochelle Warner, Joshua A. Ydstie, Larry W. Wulf, Ronette Gehring, Johann F. Coetzee, Jonathan P. Mochel, Patrick J. Gorden

**Affiliations:** ^1^Department of Veterinary Diagnostic and Production Animal Medicine, Iowa State University College of Veterinary Medicine, Ames, IA, United States; ^2^Analytical Chemistry Section, Veterinary Diagnostic Laboratory, Iowa State University College of Veterinary Medicine, Ames, IA, United States; ^3^Veterinary Pharmacotherapy and Pharmacy, Department of Population Health Sciences, Utrecht University, Utrecht, Netherlands; ^4^Department of Anatomy and Physiology, Kansas State University College of Veterinary Medicine, Manhattan, KS, United States; ^5^SMART Pharmacology, Department of Biomedical Sciences, Iowa State University College of Veterinary Medicine, Ames, IA, United States

**Keywords:** meloxicam, pharmacokinetics, post-partum, NSAID, dairy

## Abstract

Lactating dairy cattle are at risk for various painful conditions throughout their life, such as lameness, parturition, mastitis, and metabolic disorders. These conditions necessitate adequate methods of analgesia to address welfare concerns through efficacious pain mitigation. As no method of analgesia has been approved for lactating dairy cattle, to date, research is necessary to determine effective pain management strategies for dairy cattle. In both the European Union and Canada, meloxicam has been approved for use in lactating dairy cattle as a methodology for pain control. The objective of this study was to characterize the pharmacokinetics of meloxicam administered orally and intravenously to lactating dairy cattle in the post-partum vs. mid-lactation period. In this parallel study design, 12 healthy, lactating Holsteins were enrolled within 24 h of freshening and randomly allocated to intravenous (0.2 mg/kg) or oral (1.0 mg/kg) meloxicam administration treatment groups. They were matched based on parity to 12, healthy cows that were considered mid-lactation [>150 days-in-milk (DIM)] to receive the same treatment. Based on meloxicam formulation, sampling times varied and plasma was collection via jugular venipuncture for 6 days. Plasma drug concentrations were evaluated using liquid chromatography coupled with mass spectroscopy and pharmacokinetic properties were evaluated using non-compartmental (i.e., statistical moments) analysis. Results indicated a decreased systemic clearance of meloxicam in post-partum relative to mid-lactation cows, which resulted in a longer half-life and increased total exposure independent of mode of administration. These results suggest a need for dose adjustments based on stage in lactation and further assessment of the impact of days-in-milk on milk withholding period.

## Introduction

The limitations in pain control in the cattle industry have significant implications for animal well-being. In dairy cattle, the most painful afflictions are lameness, parturition, mastitis, and metabolic disorders. To date, there remains no labeled pain control products for lactating dairy cattle. Therefore, there is a critical need to develop adequate strategies for pain modulation in the livestock industry. Two commonly used non-steroidal anti-inflammatory drugs (NSAIDs) for modulating painful stimuli in lactating dairy cattle are flunixin and meloxicam.

The analgesic and anti-inflammatory effects of meloxicam have been evaluated in various situations known to cause pain. Specifically, previous research has shown meloxicam to be efficacious in mitigating pain associated with castration (1), dehorning (2–5), mastitis (6), dystocia (7, 8) and diarrhea (9). Additionally, pain has been evaluated in the post-partum period with a pressure mat and showed that an altered hindlimb weight distribution is likely due to decreased pain associated with meloxicam treatment (10).

Importantly, numerous studies have evaluated the effects of administering meloxicam in the painful parturition period (8, 11, 12). Carpenter et al. (11) began the investigation with the comparison of sodium salicylate and oral tablet meloxicam administration within 12 to 36 h post-parturition in lactating dairy cattle. Most notably, both meloxicam and sodium salicylate treatment groups increased daily milk production by 4 and 3.5 kg/d, respectively, relative to placebo control. Statistically significant milk production differences were not evident until week seven of evaluation (11). Another research group evaluated the behavior, health and production effects of meloxicam after a single oral administration of 1 mg/kg BW (tablet form) relative to an empty gel capsule placebo (8). As a whole, research has shown clear downstream production benefits to the administration of meloxicam to mitigate the painful parturition period. While it may be tempting to prescribe meloxicam in the periparturient period to harvest an increased milk production, in the US, extra-label use of drugs for enhancement of milk production is illegal (13). However, if there were benefits in health parameters as have been proven by our research group (10) and others (12), then extra-label use could be prescribed provided the regulations of the Animal Medicinal Drug Use Clarification Act (AMDUCA) were followed, which included the requirement that no violative drug residues are found in the meat or milk of treated animals (13).

A rich body of literature has reported the pharmacokinetic properties of meloxicam under various formulations and dosing protocols in ruminant and pre-ruminant calves (3, 14–17). In lactating dairy cattle, pharmacokinetic differences between meloxicam oral solution and subcutaneous administration have been evaluated. Interestingly, a parity effect was evident. The parity effect is thought to be due to higher blood levels in first lactation animals, as well as, differences in metabolic and endocrine profiles due to production and growth. When controlling for treatment, first lactation animals displayed a significantly larger elimination half-life, peak concentrations (C_max_), and total systemic exposure (AUC_0−∞_) when compared with second- or greater lactation cows (18). Malreddy et al. (19) have evaluated the plasma pharmacokinetics and milk depletion profiles of co-administration of meloxicam and gabapentin in mid-lactation dairy cattle. They determined a milk withhold of 80 h for meloxicam following oral administration at 1 mg/kg. Our research group has also evaluated pharmacokinetic comparisons of milk and plasma profiles after oral tablet administration of meloxicam in lactating dairy cattle. Apparent differences were noted when comparing the pharmacokinetics of oral meloxicam between post-partum and mid-lactation dairy cows based on milk concentration time-courses. More specifically, a 210% relative bioavailability was observed in post-partum relative to mid-lactation cows. It was believed that differences in clearance per fraction of the dose absorbed and volume of distribution per fraction of the dose absorbed could be confounded by the difference in bioavailability (20). These results precipitated the need to assess absolute bioavailability in the post-partum and mid-lactation period to evaluate the effects of oral meloxicam relative to 100% bioavailable intravenous meloxicam administration.

The objective of this study was to characterize the pharmacokinetics of meloxicam administered orally and intravenously to lactating dairy cattle in the post-partum vs. mid-lactation period. We hypothesized that post-partum cows would display increased bioavailability and prolonged terminal half-life relative to mid-lactation cows independent of the mode of administration. Our null hypothesis was that bioavailability in post-partum cows was not different from mid-lactation cows, while the alternate hypothesis was that oral meloxicam bioavailability would be higher in post-partum cows than mid-lactation animals.

## Materials and Methods

### Animals

This study was completed at the Iowa State University Dairy Farm. The lactating herd consisted of ~433 animals (95% Holstein, 5% Jersey), with a 305-day mature equivalent of 11,363 kg milk, 432 kg fat, and 375 kg protein. Cows were eligible for enrollment if they had no history of meloxicam treatment in the past 30 days and were healthy prior to enrollment based on treatment history, milk production and calving ease score ≤ 2. Twelve, mixed parity post-partum Holsteins were enrolled within 24-h of freshening. They were matched on enrollment day to twelve mid-lactation Holsteins of equal parity and >150 days-in-milk. Matched pairs were randomly allocated to one of two treatment groups based on mode of administration of meloxicam. Table 1 displays animal characteristics by treatment group and by mode of administration. Parity and DIM were consistent between treatment groups and by mode of administration (Table 1).

**Table 1:** Distribution of cows matched based on parity and days in milk (DIM) after random allocation to meloxicam formulation administered.

	***n***	**Parity, #**	**DIM, d**
Mid-lactation	11	2 (1, 3)	224 (197–261)
Post-partum	13	2 (1–3)	0 (0–0)^*^
		*P* = 0.927	*P* <0.0001
Intravenous	12	2 (1–3)	9 (18–164)
Oral	12	2 (1–2)	15 (38–202)
		*P* = 0.56	*P* = 0.509

*Cows allocated to intravenous meloxicam received 0.2 mg/kg, whereas, cows receiving oral meloxicam received 1 mg/kg*.

* *Denotes that all cows were enrolled within 24 h of calving, and therefore considered 0 DIM*.

During the course of the trial, cows were housed in a free-stall barn bedded with recycled manure solids, which was standard practice at the dairy. Cows received a total mixed ration and water *ad libitum*. Ration parameters met or exceeded those recommended by the National Research Council guidelines (21). Diet remained consistent between animals. Rumen fill, demeanor, and hydration status was assessed during daily physical examination. Overall, cow housing and management met or exceeded the recommendations listed in the *Guide for Care and Use of Agricultural Animals in Research and Teaching* (22). Cows were milked three times daily at 4 A.M., 12 P.M., and 8 P.M. Iowa State University's Institutional Animal Use and Care Committee approved the research protocol prior to commencement of trial procedures (protocol number 4-17-8501-B). Animals administered meloxicam were given milk withholds of 144 h for post-partum and 96 h for mid-lactation based on literature estimates generated by Gorden et al. (20) and following on-farm protocols.

### Experimental Design and Blood Collection

Post-partum cows that met enrollment criteria were randomly allocated to one of two treatment groups within 24 h of freshening: (1) post-partum (*n* = 7) and mid-lactation (*n* = 5) cows, 0.2 mg/kg I.V meloxicam (Metacam solution, 5 mg/mL, Boehringer Ingelheim Vetmedica, Inc, St. Joseph, MO), or (2) post-partum (*n* = 6) and mid-lactation (*n* = 6), 1 mg/kg P.O meloxicam (Meloxicam tablet, 15 mg, Cadila Healthcare Ltd., India). Meloxicam tablets were administered in gelatin boluses (size #07, Torpac Inc., Fairfield, NJ) using a balling gun to place tablets in the esophagus.

Selection criteria for the post-partum groups was the same as the mid-lactation with the exception of the evaluation of eutocia. Mid-lactation cows were matched to post-partum cows based on parity. An equal distribution of L1, L2, L3+ were assigned to the study. Cows were evaluated for inclusion daily prior to the 8 A.M. milking. All cows were weighed prior to treatment. Milk was discarded for the post-partum group for 144 h and the mid-lactation group for 96 h after meloxicam administration regardless of formulation

Blood collection occurred from all cows immediately prior to the administration of meloxicam (T0). Blood was collected via venipuncture from the jugular vein into two 10 mL heparin tubes and immediately placed on ice. Following treatment, blood was collected at +5, 10, 15, 30, and 60 min, 2, 4, 8, 16, 24, 48, 72, 96, 120, and 144 h for the intravenous administered meloxicam group. Blood was collected at +4, 8, 12, 16, 20, 24, 48, 72, 96, 120, and 144 h for the orally administered meloxicam group. Blood samples were centrifuged for 20 min at 2,700 g within 30 min of sampling. Plasma was harvested and stored at −80°C until analyzed for drug concentration by liquid chromatography coupled with mass spectroscopy (LCMS/MS).

### Plasma Meloxicam Concentration Analysis

Meloxicam concentrations in plasma were determined using LCMS/MS. The method was originally described in porcine plasma and later adapted to bovine plasma (20, 23). Calibration curve correlation coefficient (*r*^2^) exceeded 0.9957 across the entire concentration range. The lower limit of detection (LLOD) was 0.4 ng/mL, and the lower limit of quantification (LLOQ) was 2 ng/mL. The accuracy and precision for the quality control (QC) samples were 97.55 and 2.7% for 15 ng/mL, 95.21 and 3.1% for 150 ng/mL, and 109.14% and 1.0% for 1,500 ng/mL, respectively.

### Pharmacokinetic Analysis

A non-compartmental pharmacokinetic approach was used to analyze plasma drug concentration-time profiles (Phoenix WinNonLin 6.4, Certara, Cary, NC, USA). Pharmacokinetic parameters analyzed included: maximum meloxicam plasma concentration (C_max_), time of maximum meloxicam concentration (T_max_), area under the meloxicam concentration-time curve to last collected time point (AUC_last_), area under the meloxicam concentration-time curve extrapolated to infinity (AUC_∞_), area under the meloxicam concentration-time curve percent extrapolated (*AUC*_*%extrapolated*_), slope of the elimination phase (λ_z_) where linear regression of the logarithmic concentration vs. time curve during the elimination phase is used to compute, meloxicam terminal half-life (T_1/2λ*z*_) where T_1/2λ*z*_ = ln(2)/λ_z_, volume of distribution (V_d_), apparent volume of distribution of meloxicam during the elimination phase (V_z_/F) where V_z_/F = Dose/(AUC_∞_ x λ_z_), mean residence time (MRT) where MRT = AUMC_∞_/AUC_∞_, clearance (CL), and meloxicam apparent systemic clearance (CL/F) where CL/F = Dose/AUC_∞_.

For analysis of meloxicam concentration, the first value below the LLOQ was inferred to be LLOQ/2, and succeeding data points were excluded from the analysis. A linear-log trapezoidal rule was used to estimate the area under the meloxicam time curves. Summary statistics on the individual pharmacokinetic parameters were performed thereafter to derive the geometric mean and 95% confidence intervals. Geometric mean is preferred to arithmetic mean due to the small size and the moderately large quantity of data below the analytical quantification limit.

Bioavailability (F) for post-partum and mid-lactation treatment groups was calculated using:

$$\begin{array}{l} F=\frac{AUC_{oral}\;\times \;Dose_{IV}\;}{AUC_{IV}\;\times \;Dose_{oral}\;} \end{array}$$

This equation is derived from the assumption that clearance after IV and PO administration remains the same. For the aforementioned equation, AUC_∞_ was used for both treatment groups.

The global extraction ratio (*E*) was calculated according to the following formula:

$$\begin{array}{l} E=\frac{CL_{Total}}{Q_{Cardiac}} \end{array}$$

The global extraction ratio is a numerical value between 0 and 1, which represents the proportion of drug cleared from a single passage through the clearing organ (24). For the aforementioned equation, *CL*_*Total*_ is the absolute systemic clearance of meloxicam, while *Q*_*Cardiac*_ is the estimated cardiac blood flow. One assumption is that the concentration of drug in whole blood is equal to that of drug in plasma. Another assumption is that cardiac output blood flow can be estimated based on the allometric relationship between animal body weight and cardiac output through the equation Q_cardiac_ = 180 x BW^−0.19^ (in mL/min/kg) (25). Body weight (BW) is the estimated body weight of a Holstein cow, which was based on the mean BW of cows enrolled in this study was 671 kg.

### Statistical Analysis

Statistical analysis was performed using a commercially available software program (JMP version 14.3.0, SAS Institute Inc., Cary, NC). Single observation enrollment variables (lactation number, days in milk) were assessed using a paired *t*-test. Individual data observations to ensure equal distribution of parity and DIM were analyzed using non-parametric testing with no assumption regarding the underlying distribution between treatment groups. Non-parametric Wilcoxon Rank Sums 2-sample normal approximation was used to assess differences in individual pharmacokinetic parameters. Differences between treatment groups (post-partum vs. mid-lactation), time, and treatment by time interaction were analyzed via repeated measures using animal as a random effect nested within treatment and *F*-tests for significance of main and interaction effects. Statistical significance was established *a priori* when *P* < 0.05.

## Results

The differences in enrollment characteristics were limited to DIM when comparing post-partum and mid-lactation groups (*P* < 0.0001). The distribution of parity was equivalent between post-partum and mid-lactation due to matching (Table 1). There were no other statistical differences evident between treatment groups.

Due to a mastitis treatment, a mid-lactation cow was removed from the study. One post-partum animal could not be matched based on parity and enrollment criteria to a cow in the mid-lactation group. This resulted in a total of *n* = 11 animals in the mid-lactation treatment group and *n* = 13 in the post-partum treatment group.

**Figure 1:**
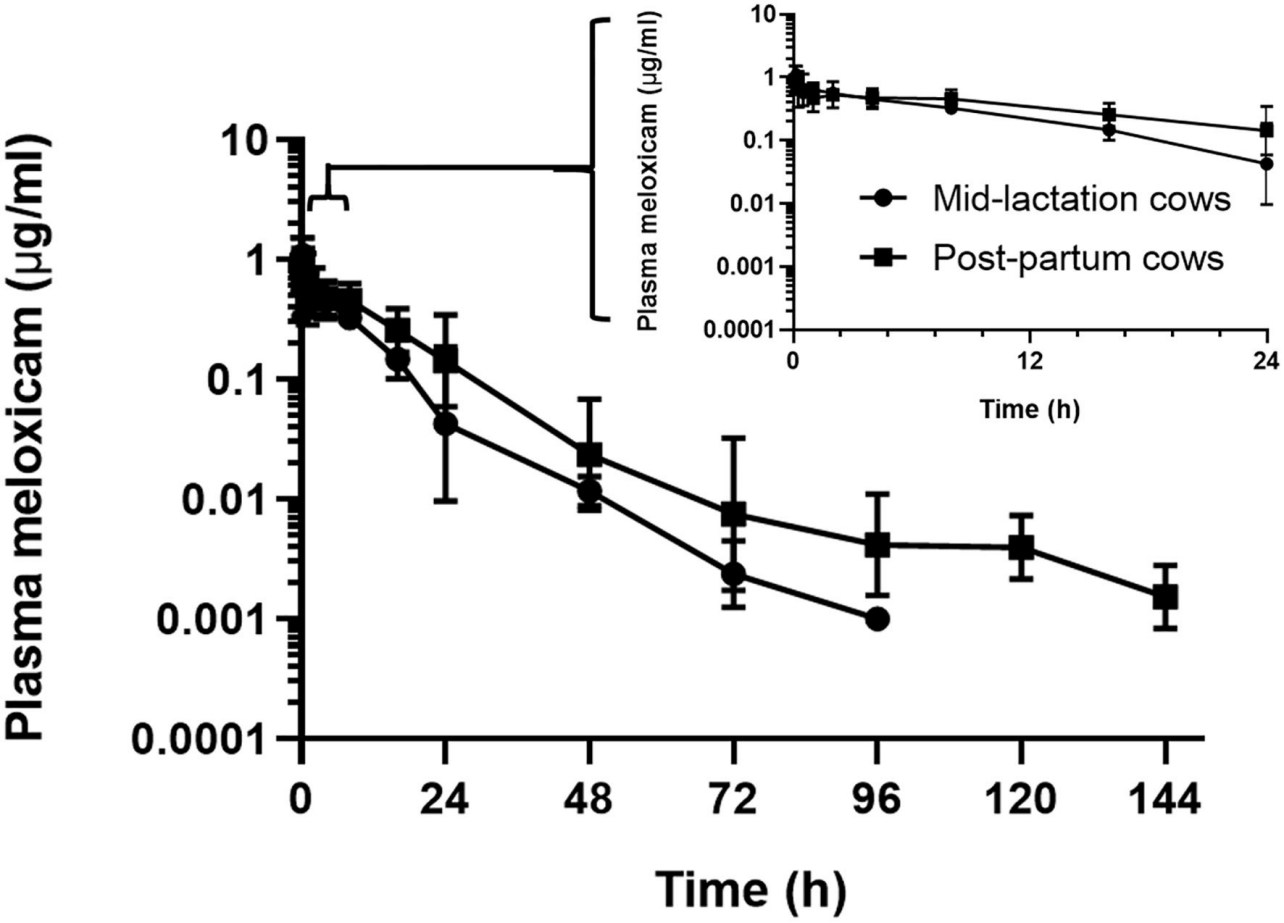
Semilogarithmic transformation of geometric mean plasma concentration (with 95% confidence interval) for post-partum (*n* = 7) and mid-lactation (*n* = 5) cows that received a single dose of intravenous meloxicam at 0.2 mg/kg. Inset contains first 24 h after meloxicam administration.

**Figure 2:**
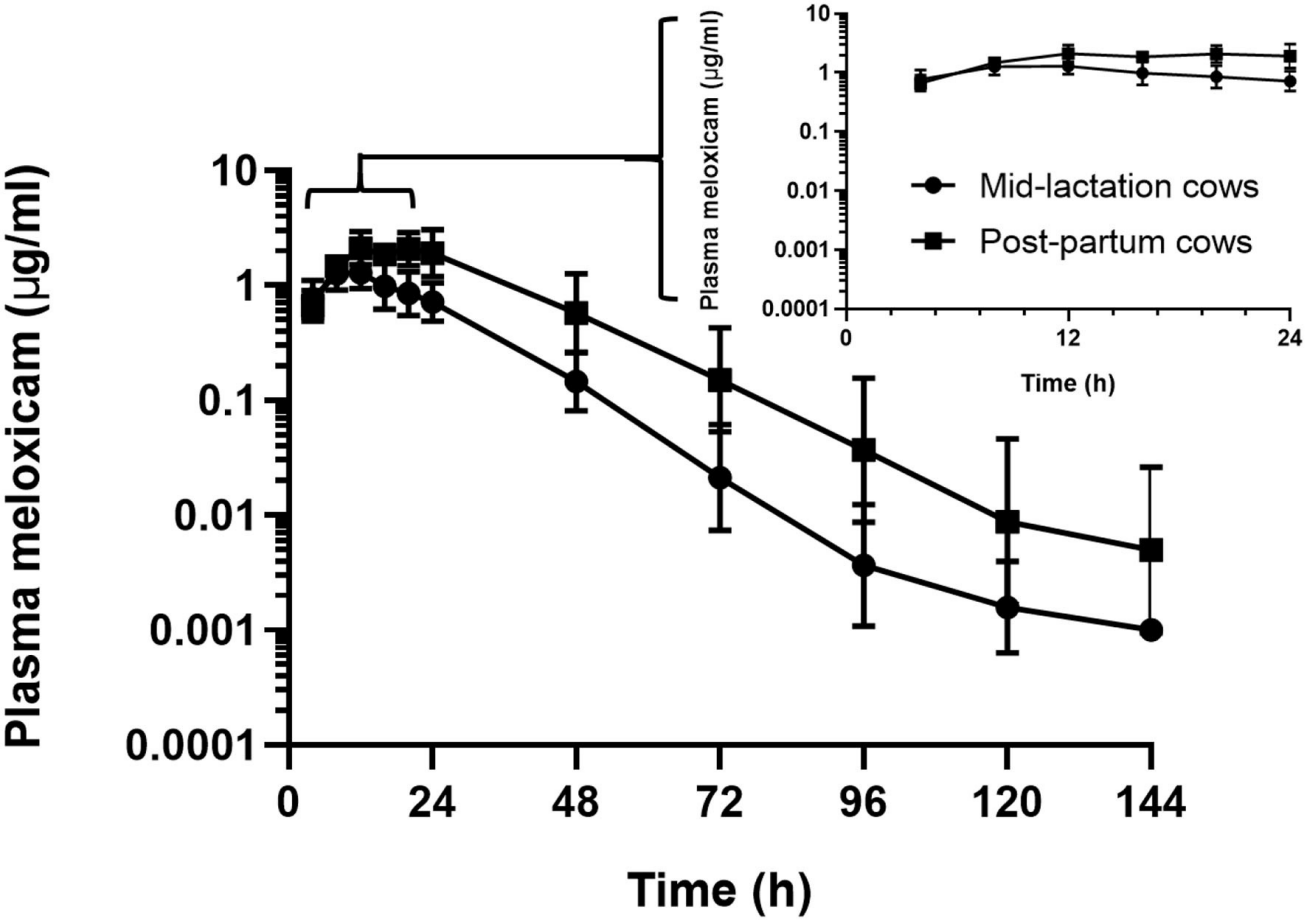
Semilogarithmic transformation of geometric mean plasma concentration (with 95% confidence interval) for post-partum (*n* = 6) and mid-lactation (*n* = 6) cows that received a single dose of oral meloxicam at 1.0 mg/kg. Inset contains first 24 h after meloxicam administration.

In the assessment of meloxicam drug concentration across time, no animal displayed meloxicam concentrations at T0. Geometric mean and 95% confidence interval disposition profiles are presented in Figure 1 for intravenous administration and Figure 2 for oral administration of meloxicam in post-partum and mid-lactation dairy cattle. Statistical differences between mid-lactation and post-partum animals were seen as early as 16 h after intravenous administration (Table 2) and oral administration (Table 3), with *P*-values of 0.0348 and 0.0131, respectively.

**Table 2:** Plasma concentrations (μg/mL) for meloxicam from seven post-partum cows compared to five mid-lactation cows that received intravenous administration of a single dose of meloxicam at 0.2 mg/kg.

**Time**	**Mid-lactation (μg/mL)**	**Post-partum (μg/mL)**	***P*-value**
5 min	0.96 (0.76–1.18)	0.88 (0.60–1.32)	1.00
10 min	1.09 (0.68–1.59)	0.84 (0.69–1.04)	0.26
15 min	0.74 (0.57–0.93)	0.69 (0.48–1.07)	0.75
30 min	0.57 (0.45–0.71)	0.64 (0.47–0.92)	0.14
60 min	0.64 (0.48–0.82)	0.42 (0.27–0.67)	0.10
2 h	0.56 (0.48–0.65)	0.50 (0.34–0.74)	1.00
4 h	0.45 (0.26–0.69)	0.46 (0.35–0.61)	0.87
8 h	0.33 (0.27–0.39)	0.48 (0.36–0.65)	0.07
16 h	0.16 (0.10–0.23)	0.31 (0.23–0.43)	0.03
24 h	0.09 (0.06–0.12)	0.20 (0.14–0.28)	0.01
48 h	0.01 (0.008–0.02)	0.05 (0.009–0.13)	0.05
72 h	< LLOQ	0.03 (0.00–0.12)	–
96 h	< LLOQ	0.007 (0.005–0.009)	–
120 h	< LLOQ	0.007^*^	–
144 h	< LLOQ	< LLOQ	–

*Results are presented as geometric means and 95% confidence interval*.

*Lower limit of quantification, LLOQ = 0.002 μg/mL. Data was analyzed with the first value below the LLOQ, replaced with $\frac{1}{2}$ LLOQ. The values below this point were discarded and here listed as < LLOQ*.

* *No 95% confidence interval display indicates detection limited to one animal*.

**Table 3:** Plasma concentrations (μg/mL) for meloxicam from six post-partum cows compared to mid-lactation cows that received oral administration of a single dose of meloxicam at 1.0 mg/kg.

**Time (h)**	**Mid-lactation (μg/mL)**	**Post-partum (μg/mL)**	***P*-value**
4	0.77 (0.53–1.09)	0.66 (0.42–0.97)	0.81
8	1.27 (0.92–1.73)	1.48 (1.21–1.80)	0.47
12	1.29 (0.93–1.75)	2.11 (1.49–2.92)	0.07
16	0.99 (0.63–1.52)	1.86 (1.54–2.24)	0.01
20	0.85 (0.51–1.32)	2.08 (1.46–2.88)	0.008
24	0.72 (0.44–1.09)	1.91 (0.96–3.26)	0.008
48	0.15 (0.07–0.28)	0.57 (0.22–1.23)	0.01
72	0.03 (0.00–0.10)	0.15, (0.03–0.42)	0.04
96	0.01 (0.00–0.14)	0.06 (0.00–0.21)	0.33
120	0.006^*^	0.03 (0.00–0.17)	0.37
144	LLOQ	0.03^*^	–

*Results are presented as geometric means and 95% confidence intervals*.

*Lower limit of quantification, LLOQ = 0.002 μg/mL. Data was analyzed with the first value below the LLOQ, replaced with $\frac{1}{2}$ LLOQ. The values below this point were discarded and here listed as < LLOQ*.

* *No 95% confidence interval display indicates detection limited to one animal*.

Summary plasma pharmacokinetic parameters for meloxicam are displayed in two tables, which are separated by intravenous (Table 4) and oral (Table 5) administration to compare the differences between treatment groups based on non-compartmental analysis. For all treatment groups, the estimated AUC_%extrapolated_ was below the 20% acceptable limit. Overall, the peak plasma concentration (C_max_) estimated after intravenous administration was 1.22 (0.92–1.55) μg/mL and 1.06 (0.84–1.34) μg/mL for mid-lactation and post-partum treatment groups, respectively. This occurred at 0.13 (0.08–0.19) h and 0.23 (0.08–4.01) h, respectively. Due to the use of non-compartmental analysis C_0_ could not be extrapolated. After oral administration, meloxicam peak concentrations were estimated at 1.45 (1.12–1.88) μg/mL and 2.61 (1.79–3.67) μg/mL at 10.48 (8.50–12.83) h and 16.75 (12.25–22.42) h for mid-lactation and post-partum groups, respectively.

**Table 4:** Plasma pharmacokinetic parameters for meloxicam from seven post-partum cows compared to five mid-lactation cows intravenously administered a single dose of meloxicam at 0.2 mg/kg.

**IV**	**Mid-lactation**	**Post-partum**	***P*-value**
C_max_ (μg/mL)	1.22 (0.92–1.55)	1.06 (0.84–1.34)	0.26
T_max_ (h)	0.13 (0.08–0.19)	0.23 (0.08–4.01)	0.73
V_d_ (L/kg)	0.29 (0.24–0.35)	0.31 (0.19–0.48)	0.94
CL (L/kg/h)	0.03 (0.02–0.03)	0.01 (0.009–0.02)	0.009
AUC_∞_ (h x μg/mL)	8.26 (6.62–10.10)	16.30 (6.18–31.75)	0.009
AUC_%extrapolated_	1.65 (1.29–2.06)	1.68 (−11.13–29.84)	0.14
λ_z_ (h^−1^)	0.08 (0.08–0.09)	0.04 (0.03–0.07)	0.006
AUMC_∞_ (h x μg/mL)	93.33 (72.00–118.95)	393.75 (115.22–3908.18)	0.009
MRT_∞_ (h)	11.30 (10.44–12.19)	24.16 (12.23–85.36)	0.006
T_1/2_ (h)	8.23 (7.90–8.58)	17.31 (8.59–64.05)	0.006
E	0.008 (0.006–0.01)	0.004 (0.003–0.006)	0.009

*Results are presented in geometric means and 95% confidence intervals. P-values are based on non-parametric Wilcoxon Rank Sums 2-sample normal approximation*.

*Parameters include maximum plasma concentration (C_max_), time of C_max_ (T_max_), area under the curve extrapolated to infinity (AUC_∞_), area under the first momentum curve extrapolated to infinity (AUMC_∞_), area under the curve percent extrapolated (AUC_%extrapolated_), slope of terminal phase (λ_z_), terminal half-life (T_1/2_), volume of distribution (V_d_), mean residence time (MRT_∞_), and clearance (CL)*.

**Table 5:** Plasma pharmacokinetic parameters for meloxicam from six post-partum cows matched to mid-lactation cows orally administered a single dose of meloxicam at 1.0 mg/kg.

**Oral**	**Mid-lactation**	**Post-partum**	***P*-value**
C_max_ (μg/mL)	1.45 (1.12–1.88)	2.61 (1.79–3.67)	0.02
T_max_ (h)	10.48 (8.50–12.83)	16.75 (12.25–22.42)	0.02
V_z_/F (L/kg)	0.39 (0.27–0.60)	0.22 (0.17–0.33)	0.02
CL/F (L/kg/h)	0.03 (0.02–0.04)	0.01 (0.007–0.019)	0.008
AUC_∞_ (h x μg/mL)	36.01 (24.29–51.02)	82.82 (50.55–126.77)	0.008
AUC_%extrapolated_	0.60 (−0.09–1.89)	0.47 (0.17–0.89)	0.69
λ_z_ (h^−1^)	0.07 (0.06–0.08)	0.06 (0.05–0.07)	0.05
AUMC_∞_ (h x μg/mL)	733.95 (394.66–1194.39)	2287.61 (981.54–4354.35)	0.008
MRT_∞_ (h)	20.38 (17.16–24.02)	27.62 (21.56–34.90)	0.05
T_1/2_ (h)	9.55 (8.26–10.99)	12.28 (9.60–15.42)	0.05
F (%)	87.2	101.6	–^*^

*Results are presented in geometric means and range. P-values are based on non-parametric Wilcoxon Rank Sums 2-sample normal approximation*.

*Parameters include maximum plasma concentration (C_max_), time of C_max_ (T_max_), area under the curve extrapolated to infinity (AUC_∞_), area under the curve percent extrapolated (AUC_%extrapolated_), area under the first momentum curve to infinity (AUMC_∞_), slope of terminal phase (λ_z_), terminal half-life (T_1/2_), volume of distribution per fraction of drug absorbed (V_z_/F), mean residence time (MRT_∞_), clearance per fraction of drug absorbed (CL/F), and absolute bioavailability (F_abs_)*.

* *Statistical comparisons could not be made between treatment groups due individual animals receiving a single treatment and therefore clearance cannot be assumed to be consistent*.

Independent of formulation, post-partum cattle that were administered meloxicam displayed significantly higher AUC_∞_ after both intravenous and oral administration (*P* = 0.0094 and 0.0082, respectively) (Tables 4, 5). Importantly, a significant 1.92-fold decrease in absolute clearance for meloxicam was noted between mid-lactation and post-partum cows (*P* = 0.0094) (Table 4). The estimated oral bioavailability of meloxicam was 87.2% for mid-lactation and 101.6% for post-partum cattle (Table 5). This is on average a 14.4% gain in the proportion of meloxicam that reaches systemic circulation in cattle in the post-partum period. Lastly, the mid-lactation and post-partum global extraction ratio of meloxicam for intravenous administration was 0.008 (0.006–0.10) and 0.004 (0.003–0.006), respectively (Table 4). These results confirm that meloxicam is acting as a low extraction ratio drug in both post-partum and mid-lactation conditions.

## Discussion

Lactating dairy cattle undergo painful stimuli as they transition into lactation (8, 11, 12). Yet, the lack of approved analgesics for lactating dairy cattle raises welfare concerns. Additionally, understanding the pharmacokinetic properties of NSAIDs, like meloxicam, is necessary for judicious extra-label drug use of pain mitigation strategies in dairy cattle.

The previous work by Carpenter et al. (11) administered meloxicam in tablet form at a single dose of 1 mg/kg BW and the control group received a placebo bolus with water drench. The investigators evaluated circulating levels of glucose, β-hydroxybutyrate (BHB), free fatty acids, haptoglobin, paraoxonase, milk production, body condition score, reproductive status, and retention in the herd. They found that meloxicam-treated animals displayed an increase in plasma glucose concentration relative to control and alternate drug treatment groups. The sodium salicylate treatment group displayed a decrease in plasma BHB, but an increase in haptoglobin compared to control cows. In the 365-day window that cows were assessed, control subjects were removed more quickly from the herd than meloxicam-treated cows. The daily milk production increases due to meloxicam and sodium salicylate treatment groups were 4 and 3.5 kg/d, respectively. This was not statistically significant until week seven of evaluation, indicating an influence on peak milk (11).

Swartz et al. (8) compared meloxicam effects during and after calving vs. control. Cows were further evaluated for calving difficulty, using > 70 min as the breakpoint for dystocia event. Milk yields were evaluated for 15 weeks, as well as, behavioral events tracked by an accelerometer. The results revealed that dystocic animals that received meloxicam were less active than the control group. The authors hypothesized that meloxicam reduced activity and inflammation, which allowed animals that were experiencing more chronic pain due to the dystocia event, the ability to rest. They found no treatment effect for health parameters, including rectal temperature and clinical disease events. Most strikingly, a 6.8 kg/d milk yield increase was seen in cows administered meloxicam prior to an eutocic calving relative to control (*P* < 0.05). Both eutocic and dystocic calvings that were administered meloxicam prior to parturition displayed higher milk fat, protein and lactose than the control (8).

More recently, Shock et al. (12) evaluated the impact of meloxicam oral suspension (1 mg/kg BW) when administered at parturition on milk production and health. This large sample set in Canada showed a 0.64 kg/d increase in milk yield over the first three test days (90–120 days in milk), 0.75 times risk ratio of subclinical mastitis and 0.46 times risk ratio of cull or death rate within the first 60 DIM. The authors in alternative studies suggested that differences in total effect on milk production in the literature was likely due to the majority of effect occurring at peak lactation (11). Both Swartz et al. (8) and Carpenter et al. (11) assessed >weeks, whereas, Shock et al. (12) assessed the first 120 DIM, which has likely influenced the differences in production effects reported.

Our group has previously noted that meloxicam persisted at higher concentrations in the plasma for longer in post-partum vs. mid-lactation dairy cattle following oral administration (20). This finding was confirmed in the current study. In our previous work, we hypothesized that the difference was due to an increased bioavailability of the drug in post-partum cows (20). To test this hypothesis, the current study was designed to compare the pharmacokinetics of meloxicam between these two groups following both intravenous and oral administration. The results indicated a lower clearance in post-partum vs. mid-lactation cows, independent of the mode of administration, resulting in a longer estimated terminal half-life and increased systemic exposure in post-partum cows. The bioavailability determined in this study is consistent with the literature showing that meloxicam is extensively absorbed after oral dosing (14). The difference in bioavailability between treatment groups are due to absolute clearance differences between post-partum and mid-lactation cows. The clearance in post-partum was approximately half compared to mid-lactation cows. Accordingly, the systemic exposure to meloxicam in post-partum cows was doubled. Meloxicam is a low-extraction ratio drug (*E* < 0.3) and, like many other NSAIDs, is primarily eliminated by the liver. Less than 5% of unchanged drug is excreted through the renal system (26). The results displayed a global extraction ratio of meloxicam for intravenous administration as 0.008 (0.006–0.01) and 0.004 (0.003–0.006) for mid-lactation and post-partum treatment groups, respectively. These results confirm that meloxicam is a low extraction ratio drug in both post-partum and mid-lactation conditions. Extraction ratio for meloxicam has been previously reported in guinea pigs, as 0.0087, which is consistent with the mid-lactation treatment group (27). Though there is a substantial difference in the hepatic blood flow between post-partum and mid-lactation cows (28), under the assumptions made by the Well-Stirred model of hepatic drug clearance, changes in blood flow have little impact on clearance of low-extraction ratio drug (29). The Well-Stirred model can be reduced to two factors that can influence hepatic clearance of meloxicam under the assumption of a low extraction ratio drug. The first being the unbound fraction or free fraction and alternatively, the intrinsic clearance could be decreased. Therefore, clearance of drugs that have a low extraction ratio are determined by the following equation:

$$\begin{array}{l} CL_{hepatic}=\;F_{unbound}\;\times \;CL_{intrinsic} \end{array}$$

Possible explanations for the observed decreased hepatic clearance in our work include a decrease of the unbound fraction (F_unbound_) of the drug to plasma proteins, such as albumin availability, or changes in protein binding interaction. Decreased hepatic intrinsic clearance (CL_intrinsic_) could have been due to decreased metabolic enzymes. Future work on this topic should evaluate protein binding capacity and hepatic enzymatic function in the post-partum compared to mid-lactation animals.

Though the overarching objective was not to determine efficacy to evaluate concentration-effect relationships, a possible limitation to this study design was the void of detection of bound vs. unbound fraction of meloxicam. We believe that if differences in protein binding were present, they would be reflective in volume of distribution following intravenous administration. Additionally, concurrent evaluation of meloxicam milk concentrations would have strengthened the ability to assess pharmacokinetics across sample types.

## Conclusion

In conclusion, differences in meloxicam bioavailability were evident between mid-lactation and post-partum dairy cattle. Both the 33% decrease in absolute clearance and ~2-fold apparent increase of meloxicam systemic exposure in post-partum cows contributed to the increase oral bioavailability. Both clearance and area under the curve directly impact bioavailability. This reduction in clearance may necessitate a longer withdrawal time when administered in the post-partum period vs. mid-lactation period. Further research is necessary to determine the underlying mechanism of delayed clearance, impact on prescribed withdrawal periods and assessment into efficacy of meloxicam at lower dosing in the post-partum period.

## Data Availability Statement

The datasets generated for this study can be found in the Iowa State University data sharing repository: https://www.doi.org/10.25380/iastate.12605624.

## Ethics Statement

The animal study was reviewed and approved by Iowa State University's Institutional Animal Use and Care Committee. They approved the research protocol prior to commencement of trial procedures (protocol number 4-17-8501-B).

## Author Contributions

PG, RW, JC, and RG designed the study. RW, JY, and PG carried out the animal work. RW and LW completed the laboratory analysis. RW, RG, JM, and PG carried out the data analysis. RW, JM, and PG wrote the manuscript. RW, JY, LW, RG, JC, JM, and PG reviewed, read, and approved by the manuscript. All authors contributed to the article and approved the submitted version.

## Conflict of Interest

The authors declare that the research was conducted in the absence of any commercial or financial relationships that could be construed as a potential conflict of interest.

## References

[1] Olson ME Ralston B Burwash L Matheson-Bird H Allan ND. Efficacy of oral meloxicam suspension for prevention of pain and inflammation following band and surgical castration in calves. BMC Vet Res. (2016) 12:102. 10.1186/s12917-016-0735-3 27295955 PMC4907251

[2] Heinrich A Duffield TF Lissemore KD Millman ST. The effect of meloxicam on behavior and pain sensitivity of dairy calves following cautery dehorning with a local anesthetic. J Dairy Sci. (2010) 93:2450–7. 10.3168/jds.2009-2813 20494153

[3] Coetzee JF Mosher RA KuKanich B Gehring R Robert B Reinbold JB. Pharmacokinetics and effect of intravenous meloxicam in weaned Holstein calves following scoop dehorning without local anesthesia. BMC Vet Res. (2012) 8:153. 10.1186/1746-6148-8-153 22937949 PMC3503738

[4] Allen KA Coetzee JF Edwards-Callaway LN Glynn H Dockweiler J Kukanich B. The effect of timing of oral meloxicam administration on physiological responses in calves after cautery dehorning with local anesthesia. J Dairy Sci. (2013) 96:5194–205. 10.3168/jds.2012-6251 23746590

[5] Glynn HD Coetzee JF Edwards-Callaway LN Dockweiler JC Allen KA Lubbers B. The pharmacokinetics and effects of meloxicam, gabapentin, and flunixin in postweaning dairy calves following dehorning with local anesthesia. J Vet Pharmacol Therap. (2013) 36:550–61. 10.1111/jvp.12042 23473342

[6] Fitzpatrick CE Chapinal N Petersson-Wolfe CS Devries TJ Kelton DF Duffield TF. The effect of meloxicam on pain sensitivity, rumination time, and clinical signs in dairy cows with endotoxin-induced clinical mastitis. J Dairy Sci. (2013) 96:2847–56. 10.3168/jds.2012-5855 23522672

[7] Newby NC Pearl DL LeBlanc SJ Leslie KE von Keyserlingk MAG Duffield TF. Effects of meloxicam on milk production, behavior, and feed intake in dairy cows following assisted calving. J Dairy Sci. (2013) 96:3682–8. 10.3168/jds.2012-6214 23567050

[8] Swartz TH Schramm HH Bewley JM Wood CM Leslie KE Petersson-Wolfe CS. Meloxicam administration either prior to or after parturition: effects on behavior, health, and production in dairy cows. J Dairy Sci. (2018) 101:10151–67. 10.3168/jds.2018-14657 30172394

[9] Todd CG Millman ST McKnight DR Duffield TF Leslie KE. Nonsteroidal anti-inflammatory drug therapy for neonatal calf diarrhea complex: effects on calf performance1. J Animal Sci. (2010) 88:2019–28. 10.2527/jas.2009-2340 20228238 PMC7109961

[10] Kleinhenz MD Gorden PJ Burchard M Ydstie JA Coetzee JF. Rapid communication: use of pressure mat gait analysis in measuring pain following normal parturition in dairy cows. J Animal Sci. (2018) 97:846–50. 10.1093/jas/sky450 30476107 PMC6358247

[11] Carpenter AJ Ylioja CM Vargas CF Mamedova LK Mendonça LG Coetzee JF. Hot topic: early postpartum treatment of commercial dairy cows with nonsteroidal antiinflammatory drugs increases whole-lactation milk yield. J Dairy Sci. (2016) 99:672–9. 10.3168/jds.2015-10048 26519977

[12] Shock DA Renaud DL Roche SM Poliquin R Thomson R Olson ME. Evaluating the impact of meloxicam oral suspension administered at parturition on subsequent production, health, and culling in dairy cows: A randomized clinical field trial. PLoS ONE. (2018) 13:e0209236. 10.1371/journal.pone.0209236 30540846 PMC6291144

[13] US FDA (United States Food and Drug Administration). Extra-label drug use in animals. Federal Reg. (1996) 61:57732–46.

[14] Coetzee JF KuKanich B Mosher R Allen PS. Pharmacokinetics of intravenous and oral meloxicam in ruminant calves. Vet Therap. (2009) 10:E1–8. 20425727

[15] Mosher RA Coetzee JF Cull CA Gehring R Kukanich B. Pharmacokinetics of oral meloxicam in ruminant and preruminant calves. J Vet Pharmacol Therap. (2012) 35:373–81. 10.1111/j.1365-2885.2011.01331 21883284

[16] Fraccaro E Coetzee JF Odore R Edwards-Callaway LN Kukanich B Badino P. A study to compare circulating flunixin, meloxicam and gabapentin concentrations with prostaglandin E2 levels in calves undergoing dehorning. Res Vet Sci. (2013) 95:204–11. 10.1016/j.rvsc.2013.01.018 23434065

[17] Coetzee JF Mosher RA Griffith GR Gehring R Anderson DE Kukanich B. Pharmacokinetics and tissue disposition of meloxicam in beef calves after repeated oral administration. J Vet Pharmacol Therap. (2015) 38:556–62. 10.1111/jvp.12215 25708937

[18] Shock D Roche S Olson M. A Comparative pharmacokinetic analysis of oral and subcutaneous meloxicam administered to postpartum dairy cows. Vet Sci. (2019) 6:73. 10.3390/vetsci6030073 31491858 PMC6789481

[19] Malreddy PR Coetzee JF KuKanich B Gehring R. Pharmacokinetics and milk secretion of gabapentin and meloxicam co-administered orally in Holstein-Friesian cows. J Vet Pharmacol Ther. (2013) 36:14–20. 10.1111/j.1365-2885.2012.01384.x 22372845 PMC3370063

[20] Gorden PJ Burchard M Ydstie JA Kleinhenz MD Wulf LW Rajewski SJ. Comparison of milk and plasma pharmacokinetics of meloxicam in postpartum vs. mid-lactation Holstein cows. J Vet Pharmacol Therap. (2018) 41:463–8. 10.1111/jvp.12488 29430684

[21] National Research Council Subcommittee on Dairy Cattle Nutrition. Committee on Animal Nutrition Board on Agriculture and natural Resources; Division of Earth and Life Studies. Nutrient Requirements of Dairy Cattle, 7th revised edn. Washington, DC: National Academy of Sciences. (2001).

[22] Federation of Animal Science Societies. Dairy Cattle. Guide for the Care and Use of Agricultural Animals in Teaching and Research, 3rd edn. Champaign, IL: Federation of Animal Science Societies. (2010). p. 74–88.

[23] Bates JL Karriker LA Stock ML Pertzborn KM Baldwin LG Wulf LW. Impact of transmammary-delivered meloxicam on biomarkers of pain and distress in piglets after castration and tail docking. PLoS ONE. (2014) 9:e113678. 10.1371/journal.pone.0113678 25437866 PMC4249978

[24] Toutain PL Bousquet-Melou A. Plasma clearance. J Vet Pharmacol Therap. (2004) 27:415–25. 10.1111/j.1365-2885.2004.00605 15601437

[25] Toutain PL Bousquet-Melou A. Bioavailability and its assessment. J Vet Pharmacol Therap. (2004) 27:455–66. 10.1111/j.1365-2885.2004.00604 15601440

[26] Verbeeck RK Blackburn JL Loewen GR. Clinical pharmacokinetics of non-steroidal anti-inflammatory drugs. Clin Pharmacokin. (1983) 8:297–331. 10.2165/00003088-198308040-00003 6352138

[27] Moeremans I Devreese M De Baere S Croubels S Hermans K. Pharmacokinetics and absolute oral bioavailability of meloxicam in guinea pigs (Cavia porcellus). Vet Anaesthes Anal. (2019) 46:548–55. 10.1016/j.vaa.2018.11.011 31153785

[28] Sangsritavong S Combs DK Sartori R Armentano LE Wiltbank MC. High feed intake increases liver blood flow and metabolism of progesterone and estradiol-17β in dairy cattle. J Dairy Sci. (2002) 85:2831–42. 10.3168/jds.S0022-0302(02)74370-1 12487450

[29] Yang J Jamei M Yeo KR Rostami-Hodjegan A Tucker GT. Misuse of the well-stirred model of hepatic drug clearance. Drug Metab Disposit. (2007) 35:501–2. 10.1124/dmd.106.013359 17325025

