# Corrigendum: Comparative Pharmacokinetics of Meloxicam Between Healthy Post-partum vs. Mid-lactation Dairy Cattle

**DOI:** 10.3389/fvets.2021.665021

**Published:** 2021-05-26

**Authors:** Rochelle Warner, Joshua A. Ydstie, Larry W. Wulf, Ronette Gehring, Johann F. Coetzee, Jonathan P. Mochel, Patrick J. Gorden

**Affiliations:** ^1^Department of Veterinary Diagnostic and Production Animal Medicine, Iowa State University College of Veterinary Medicine, Ames, IA, United States; ^2^Analytical Chemistry Section, Veterinary Diagnostic Laboratory, Iowa State University College of Veterinary Medicine, Ames, IA, United States; ^3^Veterinary Pharmacotherapy and Pharmacy, Department of Population Health Sciences, Utrecht University, Utrecht, Netherlands; ^4^Department of Anatomy and Physiology, Kansas State University College of Veterinary Medicine, Manhattan, KS, United States; ^5^SMART Pharmacology, Department of Biomedical Sciences, Iowa State University College of Veterinary Medicine, Ames, IA, United States

**Keywords:** meloxicam, pharmacokinetics, post-partum, NSAID, dairy

In the original article, there was a mistake in the legend for **Tables 2** and 4 as published. **In these tables, the number of cows in each group were stated incorrectly**. The correct legend appears below.

**Table 4:** Plasma pharmacokinetic parameters for meloxicam from seven post-partum cows compared to five mid-lactation cows intravenously administered a single dose of meloxicam at 0.2 mg/kg.

**IV**	**Mid-lactation**	**Post-partum**	***P*-value**
C_max_ (μg/mL)	1.22 (0.92–1.55)	1.06 (0.84–1.34)	0.26
T_max_ (h)	0.13 (0.08–0.19)	0.23 (0.08–4.01)	0.73
V_d_ (L/kg)	0.29 (0.24–0.35)	0.31 (0.19–0.48)	0.94
CL (L/kg/h)	0.03 (0.02–0.03)	0.01 (0.009–0.02)	0.009
AUC_∞_ (h x μg/mL)	8.26 (6.62–10.10)	16.30 (6.18–31.75)	0.009
AUC_%extrapolated_	1.65 (1.29–2.06)	1.68 (−11.13–29.84)	0.14
λ_z_ (h^−1^)	0.08 (0.08–0.09)	0.04 (0.03–0.07)	0.006
AUMC_∞_ (h x μg/mL)	93.33 (72.00–118.95)	393.75 (115.22–3908.18)	0.009
MRT_∞_ (h)	11.30 (10.44–12.19)	24.16 (12.23–85.36)	0.006
T_1/2_ (h)	8.23 (7.90–8.58)	17.31 (8.59–64.05)	0.006
E	0.008 (0.006–0.01)	0.004 (0.003–0.006)	0.009

*Results are presented in geometric means and 95% confidence intervals. P-values are based on non-parametric Wilcoxon Rank Sums 2-sample normal approximation*.

*Parameters include maximum plasma concentration (C_max_), time of C_max_ (T_max_), area under the curve extrapolated to infinity (AUC_∞_), area under the first momentum curve extrapolated to infinity (AUMC_∞_), area under the curve percent extrapolated (AUC_%extrapolated_), slope of terminal phase (λ_z_), terminal half-life (T_1/2_), volume of distribution (V_d_), mean residence time (MRT_∞_), and clearance (CL)*.

**Table 2**. Plasma concentrations (μg/mL) for meloxicam from seven post-partum cows compared to five mid-lactation cows that received intravenous administration of a single dose of meloxicam at 0.2 mg/kg. Results are presented as geometric means and 95% confidence interval.

Table 4. Plasma pharmacokinetic parameters for meloxicam from seven post-partum cows compared to five mid-lactation cows intravenously administered a single dose of meloxicam at 0.2 mg/kg. Results are presented in geometric means and 95% confidence intervals. *P*-values are based on non-parametric Wilcoxon Rank Sums 2-sample normal approximation.

In the original article, there was a mistake in Table 4 as published. **In this table, the units for V**_**d**_ **should have been L/kg**. The corrected Table 4 appears below.

In the original article, there was a mistake in Table 5 as published. **In this table, the units for V**_**z**_**/F should have been L/kg and the values for V**_**z**_**/F were incorrectly reported as mL/kg**. The corrected Table 5 appears below.

**Table 5:** Plasma pharmacokinetic parameters for meloxicam from six post-partum cows matched to mid-lactation cows orally administered a single dose of meloxicam at 1.0 mg/kg.

**Oral**	**Mid-lactation**	**Post-partum**	***P*-value**
C_max_ (μg/mL)	1.45 (1.12–1.88)	2.61 (1.79–3.67)	0.02
T_max_ (h)	10.48 (8.50–12.83)	16.75 (12.25–22.42)	0.02
V_z_/F (L/kg)	0.39 (0.27–0.60)	0.22 (0.17–0.33)	0.02
CL/F (L/kg/h)	0.03 (0.02–0.04)	0.01 (0.007–0.019)	0.008
AUC_∞_ (h x μg/mL)	36.01 (24.29–51.02)	82.82 (50.55–126.77)	0.008
AUC_%extrapolated_	0.60 (−0.09–1.89)	0.47 (0.17–0.89)	0.69
λ_z_ (h^−1^)	0.07 (0.06–0.08)	0.06 (0.05–0.07)	0.05
AUMC_∞_ (h x μg/mL)	733.95 (394.66–1194.39)	2287.61 (981.54–4354.35)	0.008
MRT_∞_ (h)	20.38 (17.16–24.02)	27.62 (21.56–34.90)	0.05
T_1/2_ (h)	9.55 (8.26–10.99)	12.28 (9.60–15.42)	0.05
F (%)	87.2	101.6	–^*^

*Results are presented in geometric means and range. P-values are based on non-parametric Wilcoxon Rank Sums 2-sample normal approximation*.

*Parameters include maximum plasma concentration (C_max_), time of C_max_ (T_max_), area under the curve extrapolated to infinity (AUC_∞_), area under the curve percent extrapolated (AUC_%extrapolated_), area under the first momentum curve to infinity (AUMC_∞_), slope of terminal phase (λ_z_), terminal half-life (T_1/2_), volume of distribution per fraction of drug absorbed (V_z_/F), mean residence time (MRT_∞_), clearance per fraction of drug absorbed (CL/F), and absolute bioavailability (F_abs_)*.

* *Statistical comparisons could not be made between treatment groups due individual animals receiving a single treatment and therefore clearance cannot be assumed to be consistent*.

The authors apologize for this error and state that this does not change the scientific conclusions of the article in any way. The original article has been updated.

